# A Short Practice of Midwifery

**Published:** 1899-06

**Authors:** 


**A Short Practice of Midwifery.** By HENRY JELLETT, M.D. Pp. xx., 323. London: J. & A. Churchill. 1897.

In this excellent compendium of the essentials of midwifery practice, the author commences with a well-written and precise chapter on aseptic principles and their practical application to midwifery. With reference to the principles laid down criticism is needless; the details concerning which some will disagree with the author are not important so long as the general principles underlying their application are correctly understood and carried out.

The chapter on abdominal palpation contains many very valuable hints on this important subject. The careful and precise explanation of manipulations gives no excuse for misunderstanding or mistake.

One or two practical points will meet with adverse criticism. In the treatment of accidental hemorrhage the author recommends plugging the vagina tightly; he brings good reason and authority for this method, which is not universally accepted as correct, but a view which is backed by the high authority of the Rotunda school must be received with a certain amount of deference. Had he added plugging the cervix to plugging the vagina, there would have been general agreement with him.

Dr. Jellett states that in the indications for the application of forceps, the head remaining four hours above the brim in the second stage is not of necessity an indication. We have known extensive sloughing follow less delay; with weak pains no doubt delay is harmless, but with strong pains danger is its early result. In the treatment of eclampsia he disapproves of the chloral and chloroform treatment, and prefers the hypodermic administration of morphia: here again he is supported by his school, and although we should object to the adoption of any hard and fast rule in the treatment of these cases, this opinion, enforced as it is by favourable experience, should be accepted with due regard.

The tables at the end referring to the work of the Rotunda during several years are very interesting and well worth study.

